# Intra-Arterial Transplantation of Allogeneic Mesenchymal Stem Cells Mounts Neuroprotective Effects in a Transient Ischemic Stroke Model in Rats: Analyses of Therapeutic Time Window and Its Mechanisms

**DOI:** 10.1371/journal.pone.0127302

**Published:** 2015-06-15

**Authors:** Atsuhiko Toyoshima, Takao Yasuhara, Masahiro Kameda, Jun Morimoto, Hayato Takeuchi, Feifei Wang, Tatsuya Sasaki, Susumu Sasada, Aiko Shinko, Takaaki Wakamori, Mihoko Okazaki, Akihiko Kondo, Takashi Agari, Cesario V. Borlongan, Isao Date

**Affiliations:** 1 Department of Neurological Surgery, Okayama University Graduate School of Medicine, Dentistry and Pharmaceutical Sciences, 2-5-1, Shikata-cho, Kita-ku, Okayama, 700–8558, Japan; 2 Center for Innovative and Translational Medicine, Kochi University Medical School, Kohasu, Oko-cho, Nankoku, Kochi, 783–8505, Japan; 3 Department of Neurosurgery, University of South Florida College of Medicine, 12901 Bruce B Downs Blvd, Tampa, Florida, 33612, United States of America; National University of Singapore, SINGAPORE

## Abstract

**Objective:**

Intra-arterial stem cell transplantation exerts neuroprotective effects for ischemic stroke. However, the optimal therapeutic time window and mechanisms have not been completely understood. In this study, we investigated the relationship between the timing of intra-arterial transplantation of allogeneic mesenchymal stem cells (MSCs) in ischemic stroke model in rats and its efficacy in acute phase.

**Methods:**

Adult male Wistar rats weighing 200 to 250g received right middle cerebral artery occlusion (MCAO) for 90 minutes. MSCs (1×10^6^cells/ 1ml PBS) were intra-arterially injected at either 1, 6, 24, or 48 hours (1, 6, 24, 48h group) after MCAO. PBS (1ml) was intra-arterially injected to control rats at 1 hour after MCAO. Behavioral test was performed immediately after reperfusion, and at 3, 7 days after MCAO using the Modified Neurological Severity Score (mNSS). Rats were euthanized at 7 days after MCAO for evaluation of infarct volumes and the migration of MSCs. In order to explore potential mechanisms of action, the upregulation of neurotrophic factor and chemotactic cytokine (bFGF, SDF-1α) induced by cell transplantation was examined in another cohort of rats that received intra-arterial transplantation at 24 hours after recanalization then euthanized at 7 days after MCAO for protein assays.

**Results:**

Behavioral test at 3 and 7 days after transplantation revealed that stroke rats in 24h group displayed the most robust significant improvements in mNSS compared to stroke rats in all other groups (p’s<0.05). Similarly, the infarct volumes of stroke rats in 24h group were much significantly decreased compared to those in all other groups (p’s<0.05). These observed behavioral and histological effects were accompanied by MSC survival and migration, with the highest number of integrated MSCs detected in the 24h group. Moreover, bFGF and SDF-1α levels of the infarcted cortex were highly elevated in the 24h group compared to control group (p’s<0.05).

**Conclusions:**

These results suggest that intra-arterial allogeneic transplantation of MSCs provides post-stroke functional recovery and reduction of infarct volumes in ischemic stroke model of rats. The upregulation of bFGF and SDF-1α likely played a key mechanistic role in enabling MSC to afford functional effects in stroke. MSC transplantation at 24 hours after recanalization appears to be the optimal timing for ischemic stroke model, which should guide the design of clinical trials of cell transplantation for stroke patients.

## Introduction

Ischemic stroke is a major cause of morbidity and mortality, with only a few therapeutic options. Stem cell transplantation has emerged as a new approach of treatment for ischemic stroke [1–5]. To date, intracerebral, intravenous and intra-arterial routes of transplantation of stem cells are effective for ischemic stroke [3, 5–23]. The advantage of intracerebral transplantation is the targeted deposition of stem cells into the lesioned brain, compared to intra-arterial or intravenous transplantation resulting in majority of the cells being lodged in peripheral organs [9, 24]. However, intravascular (intra-arterial or intravenous) transplantation is less invasive than intracerebral transplantation, allowing grafting of cells without creating new injury of brain tissue, thus means less trauma to the stroke patient. Furthermore, while not locally deposited into the discrete ischemic regions, such peripheral route might guide stem cells to wider distribution into the stroke brain (albeit throughout the penumbra region). Thus, intravascular transplantation is considered as relatively simple, safe, and effective method of delivering stem cells to stroke brain [9]. To this end, intra-arterial transplantation may resolve the main problem of intravenous transplantation, with which transplanted cells must pass through the systemic and pulmonary circulation systems, ultimately many of the grafted cells being lodged into the peripheral organs and few cells reaching the injured brain [1, 9]. Recent studies indicate that intra-arterial stem cell transplantation for ischemic stroke could induce functional recovery in ischemic stroke animals [3, 8, 9, 12, 13, 16, 19, 20, 21, 25, 26], with a clinical phase I/II trial demonstrating its safety and feasibility in stroke patients [4].

Neuroprotective effects of mesenchymal stem cells (MSCs) transplantation have been demonstrated in animal models of stroke [1–3, 6, 8, 12, 14, 15, 19, 20, 23, 26–29]. MSCs can differentiate into endothelial cells or neuronal cells [2, 27], although controversial. Alternatively, the benefits of MSC transplantation has been ascribed to the secretion of neurotrophic factors or chemotactic cytokines such as vascular endothelial growth factor (VEGF), nerve growth factor (NGF), brain-derived neurotrophic factor (BDNF), basic fibroblast growth factor (bFGF) and stromal cell-derived factor-1α (SDF-1α) from transplanted MSCs [1, 2, 22, 27, 29]. In addition to the secretory function, migration of MSCs into the infarct lesion and reduction of the infarct volumes after transplantation may mediate the neuroprotective effects [1–3, 12, 27]. However, it remains unclear how the timing of intra-arterial MSC transplantation to ischemic stroke affects the functional recovery.

In this study, we examined the acute therapeutic window of intra-arterial allogeneic MSC transplantation in ischemic stroke model in adult rats. Furthermore, we investigated whether the optimal timing of transplantation was associated with the postulated neuroprotective mechanisms of stem cell therapy by focusing on the secretory function of MSCs.

## Materials and Methods

### Ethics statement

This study was conducted in accordance with the guidelines of the Institutional Animal Care and Use Committee of Okayama University Graduate School of Medicine. The protocol was specifically approved by the Institutional Animal Care and Use Committee of Okayama University Graduate School of Medicine (protocol #OKU- 2014282). Adult male Wistar rats were used in this study. For euthanasia, pentobarbital was used. MCAO was carried out under general anesthesia (1% halothane in 69% N2O and 30% O2). All efforts were made to minimize suffering.

### Animals

Adult male Wistar rats (CLEA JAPAN, Inc., Tokyo, Japan; n = 82) weighing 200 to 250g at the beginning of the experiment were used in this study, according to approved guidelines of the Institutional Animal Care and Use Committee of Okayama University. They were singly housed per cage in a temperature- and humidity-controlled room, maintained on a 12-hour light/dark cycle, with free access to food and water.

### Culture of MSCs

Rats were euthanized by sodium pentobarbital (200mg/kg) with subsequent removal of the femoral bones (n = 12). After the femoral bone marrow was flushed, cells were cultured at 2×10^4^ cells/cm^2^ in DMEM (Gibco, Cergy Pontoise, France) supplemented with 10% fetal calf serum (Gibco) and 1% (v/v) penicillin/streptomycin (Gibco). Cells were cultured at 37°C in a fully humidified atmosphere with 10% CO_2_ for 2 weeks. The medium was changed twice a week. The MSCs were isolated on the basis of their ability to adhere to the culture plates [18, 22]. After second passage, MSCs were used for transplantation.

### Transient Middle Cerebral Artery Occlusion

MCAO was carried out according to the intraluminal suture method (n = 62) [3, 12, 20]. Under general anesthesia (1% halothane in 69% N_2_O and 30% O_2_), the right common carotid artery (CCA), external carotid artery (ECA) and internal carotid artery (ICA) were exposed. Subsequently, the ECA was cut with a microscissor and a 4–0 monofilament nylon suture with silicone-coated tip (Xantopren L blue & ACTIVATOR Universal Liquid, Heraeus Kulzer GmbH & Co. KG, Hanau, Germany) was inserted through an arteriotomy of the right ECA into the right MCA. After MCAO for 90 minutes, the filament was withdrawn and ECA was ligated with 3–0 silk suture.

### Cell Labeling and Intra-arterial MSC Transplantation

**Fig 1:**
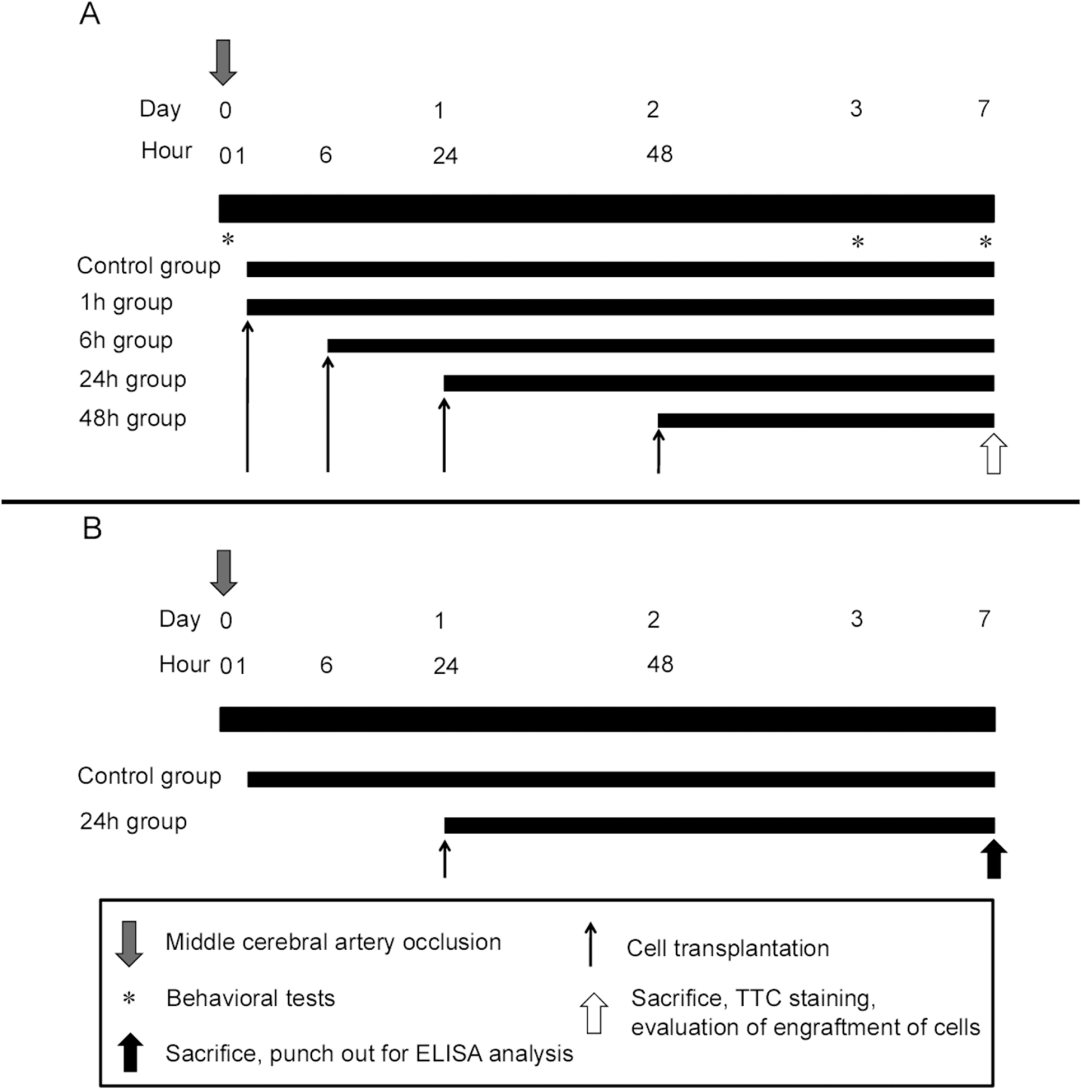
Time course. A: Schematic diagram showing overall experimental design. B: Schematic diagram showing experimental design for protein assay.

Before cell transplantation, MSCs were labeled with Q-Tracker 625 Cell Labeling kit (Q-dot nanocrystal, a red fluorescent marker; Invitrogen, Carlsbad, CA) for characterization of MSCs according to the manufacturer’s protocol. Once MSCs were cultured using Q-Tracker 625 Cell Labeling kit, Q-dot nanocrystals were delivered into the cytoplasm of MSCs. We evaluated cell labeling rate at 2 hours after cell labeling using counterstaining with 4,6-diamidino-2-phenylindole (DAPI) in vitro. Thereafter, cells were detached with 0.05% trypsin-EDTA, and one million cells were diluted with 1ml of phosphate-buffered saline (PBS). The procedures of intra-arterial transplantation in rats (n = 45) were as follows. Under general anesthesia (1% halothane in 69% N_2_O and 30% O_2_), proximal part of the right CCA was ligated by 5–0 silk suture and distal part of the right ICA was clamped by temporary clip. Subsequently, a 23G winged needle (Terumo, Tokyo, Japan) was inserted upward through an arteriotomy in the ICA. Then, ICA was declamped and cells were injected very slowly (1ml/ 2min) into ICA through the needle. After transplantation, the distal part of the ICA was ligated by 5–0 silk suture for hemostasis due to block the blood flow of ICA including an arteriotomy. Rats were divided into 4 groups by manipulating the timing of cell transplantation, in that transplantation was initiated at either 1, 6, 24, or 48 hours after recanalization. Rats in control group received 1ml PBS into the right ICA at 1 hour after recanalization as shown in Fig 1A.

### Behavioral Test

Behavioral test was performed immediately after reperfusion, and at 3 and 7 days after MCAO using the Modified Neurological Severity Score (mNSS) (n = 62). The mNSS was used to assess motor function, sensory disturbance, reflex, and balance. In this score, one score point is awarded for the inability to perform the test or for the lack of reflex. Therefore, the higher score indicates more severe injury. Neurological function was graded on a scale of 0 to 18 (normal score: 0; maximal deficit score: 18) [19, 27, 28]. Rats showed 8–12 points in the mNSS immediately after reperfusion were used in this study.

### Histological analyses

#### Measurement of the infarct volumes

Rats were euthanized under deep anesthesia using pentobarbital (100 mg/kg) with following saline perfusion and brains were quickly removed at 7 days after MCAO for 2,3,5-triphenyltetrazolium chloride (TTC) staining. Thereafter, two serial coronal sections of 2-mm thickness (at 0 and 2 mm anterior to the bregma) were prepared. Brain slices were incubated in a 0.2% solution of TTC (Kanto Chemistry Co., Tokyo, Japan) in phosphate buffered saline (PBS) at 37°C for 30 min and fixed by immersion in 4% buffered formaldehyde solution. The normal area of brain was stained dark red based on intact mitochondrial function, whereas infarct area remained unstained. Infarct area was measured using a computerized image analysis using Image J software. We evaluated infarct area ratio by the following method. Infarct area ratio = LT—(RT—RI) / LT %, where LT is the area of the left hemisphere in mm^2^, RT is the area of the right hemisphere in mm^2^ and RI is the infarct area in mm^2^. We evaluated the mean infarct area ratio of 2 sections (n = 29) [11, 30, 31].

#### Detection of integrated MSCs in vivo

At 7 days after MCAO, rats were euthanized and brains were removed. Coronal sections were cut at 30μm-thickness with a freezing microtome (-20°C) at the level of -2, 0, 2 mm anterior to the bregma. Three sections were counterstained with 4,6-diamidino-2-phenylindole (DAPI) and then were visualized with fluorescence microscopy (Keyence, Osaka, Japan) to detect MSCs labeled by Q-dot. We counted total number of nuclei of labeled MSCs in three sections, and calculated the mean number of nuclei of 3 sections. Then, we compared the mean number of nuclei of MSCs in each transplanted groups (n = 32).

### ELISA analyses

For protein assay, fresh brains from rats of control and 24h groups were quickly removed after decapitation using anesthesia with an overdose of pentobarbital (100 mg/kg, i.p.) at 7 days after MCAO. Brains were sliced at the thickness of 2 mm. The brain tissues of bilateral penumbra area of the cortex and bilateral striatum were punched out using a biopsy punch tool (3 mm-hole, Kai corporation and Kai industries co., ltd, Tokyo, Japan). Brain tissues were then homogenized in T-PER (Pierce, Rockfold, IL, USA) and centrifuged at 10,000G for 10 minutes at 4°C, and the supernatant was obtained. The bFGF and SDF-1α levels of tissues were measured by the usage of rat bFGF ELISA assay kit and mouse SDF-1α ELISA assay kit (R&D Systems, USA) (n = 8) as shown in Fig 1B.

### Statistical Analyses

Data are presented as the mean ± S.E. The data of the mNSS were statistically evaluated using repeated measures ANOVA followed by post hoc Fisher’s test. The data of histology, and ELISA were evaluated using one-factor ANOVA, followed by post hoc Fisher’s test. Statistical significance was preset at p value< 0.05.

## Results

### Detection of labeled MSCs in vitro

**Fig 2:**
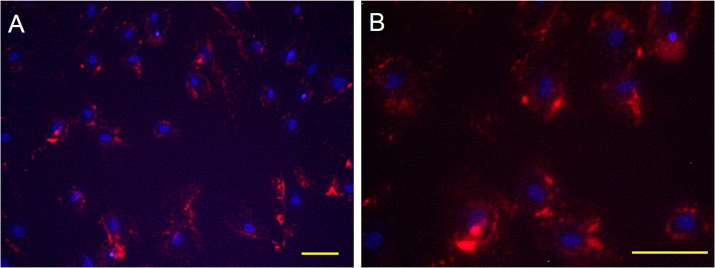
Detection of labeled MSCs in vitro. A, B: Photographs of labeled MSCs *in vitro*. Fluorescent microscope detected MSCs with red-colored cytoplasm. Scale bar: A: 40μm; B: 20μm. The cell labeling rate was 71.4±5.7% at 2 hours after cell labeling (n = 12).

After cell labeling, MSCs were visualized with fluorescent microscope on the culture plate. Cytoplasm of MSCs stained in red dye in vitro (Fig 2A and 2B). The cell labeling rate was 71.4±5.7% at 2 hours after dye treatment.

### Amelioration of neurological deficits after intra-arterial MSC transplantation

**Fig 3:**
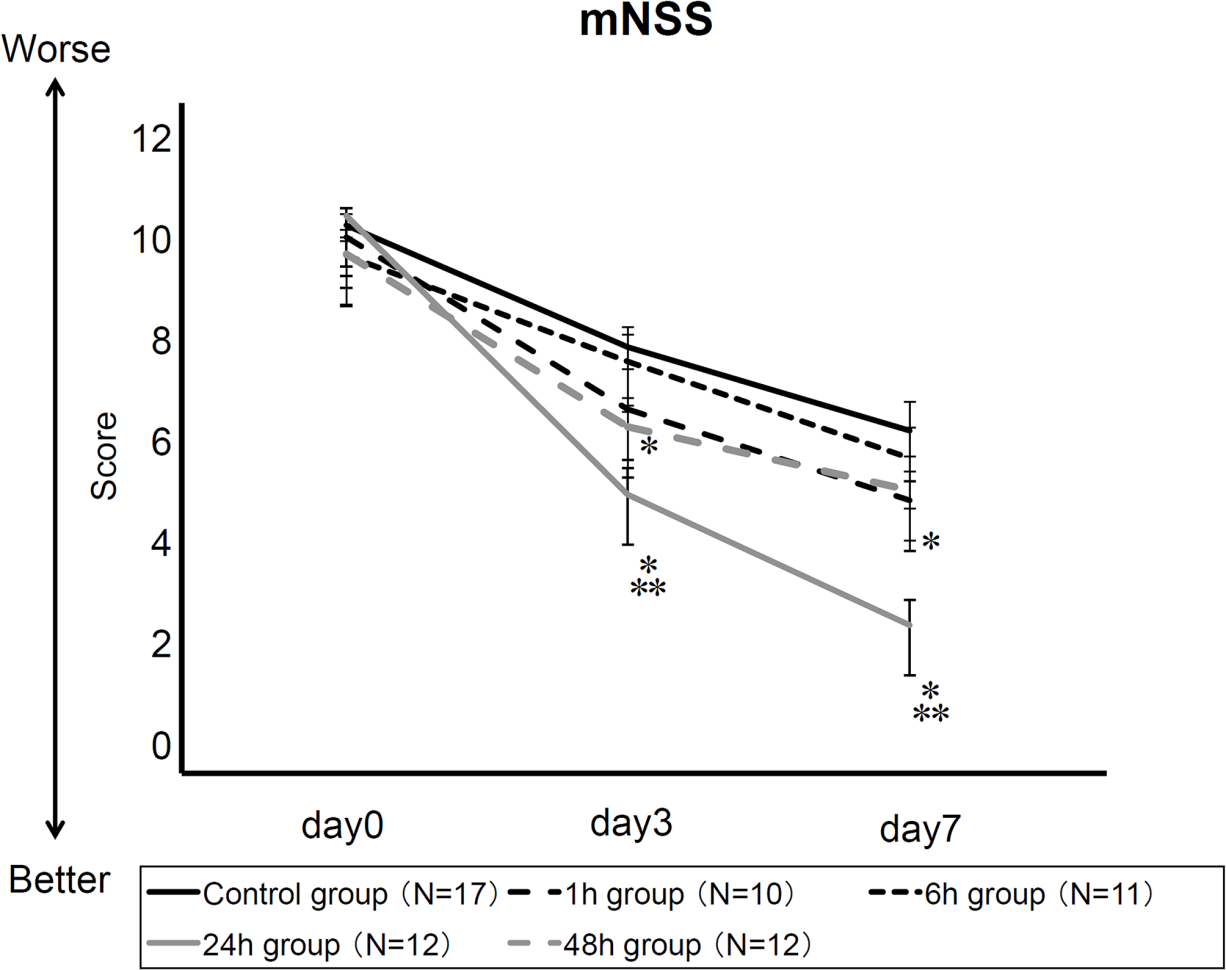
Intra-arterial MSC transplantation ameliorates stroke-induced neurological deficits. Neurological performance as revealed by mNSS shows that intra-arterial MSCs at 24h and 48h transplantation groups was better than that of control group. Rats in 24h group showed significant recovery, compared to all other groups at 3 and 7 days after MCAO (* p value<0.05 vs. control; ** p value<0.05 vs. all other groups, n = 17 in control group, 10 in 1h group, 11 in 6h group, 12 in 24h group, 12 in 48h group, respectively).

ANOVA revealed significant treatment effects in mNSS (repeated measures ANOVA; F _(4, 57)_ = 5.5, p = 0.0008). Posthoc analyses revealed that neurological function in 24h and 48h groups significantly recovered compared to control group at 3 and 7 days after MCAO (control group: 7.8±0.4 and 6.2±0.6; 1h group: 6.6±0.8 and 4.8±0.6; 6h group: 7.5±0.5 and 5.6±0.6; 24h group: 4.9±0.5 and 2.3±0.5; 48h group: 6.3±0.4 and 5.0±0.7, at 3 and 7 days after MCAO respectively, p’s<0.05, Fig 3 and S1 Data). Interestingly, stroke rats in 24h group exhibited significant improvements in neurological function compared to all other groups at 3 and 7 days after MCAO.

### Reduction of infarct volumes after intra-arterial MSC transplantation

**Fig 4:**
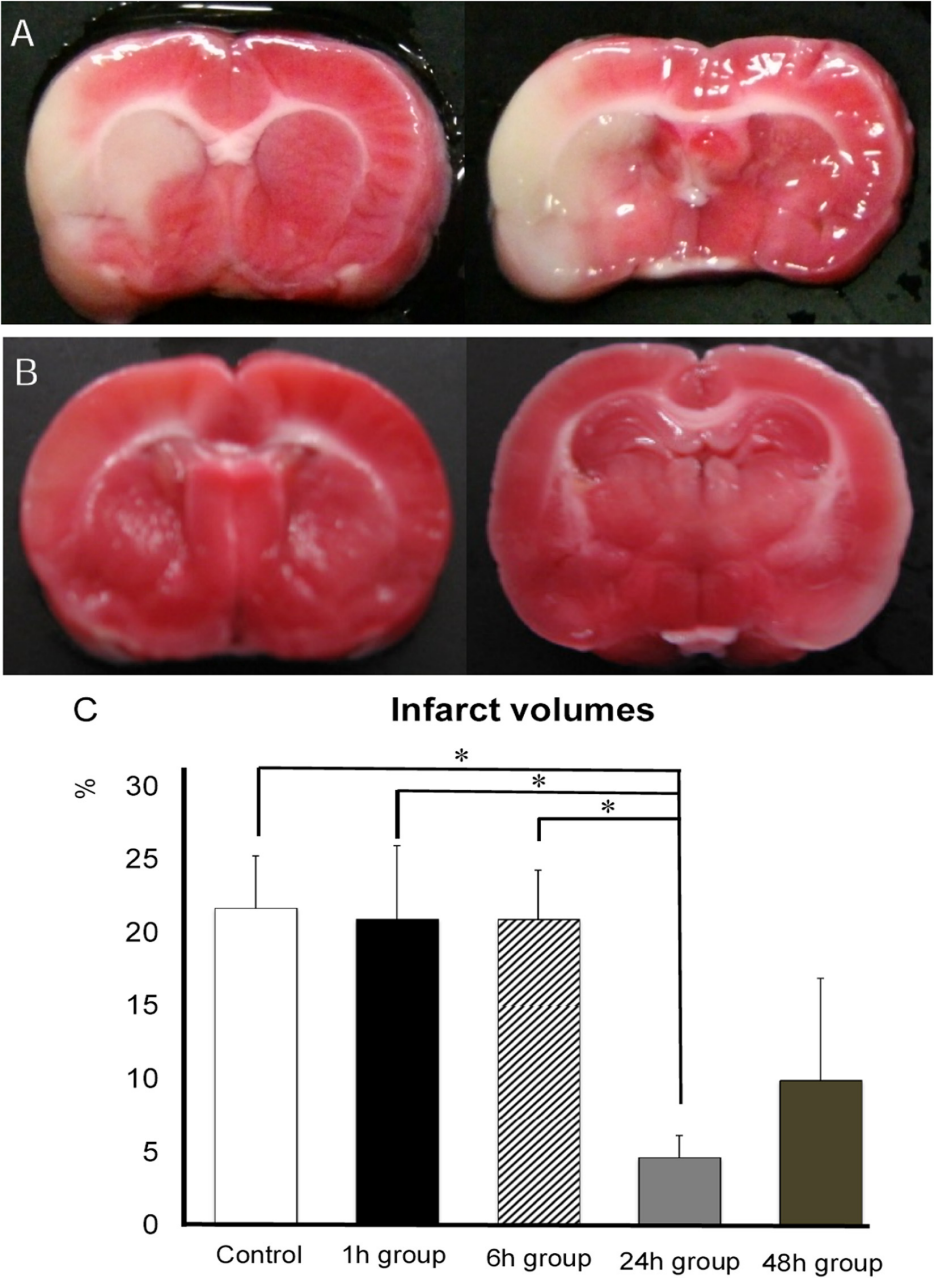
Reduction of infarct volumes after transplantation. A, B: Photographs of coronal brain sections of rats in control group (A) and 24h group (B) with 2,3,5-triphenyltetrazolium chloride (TTC) staining. C: Bar graph showing infarct volumes in each group. The infarct volumes of rats in 24h group significantly decreased compared to rats in control, 1h, and 6h groups at 7 days after MCAO (* p<0.05, n = 6 in control group, 5 in 1h group, 6 in 6h group, 6 in 24h group, 6 in 48h group, respectively). The infarct area ratio of rats in 48h group tended to decrease compared to control group, 1h group, and 6h group, but there was no statistical difference.

ANOVA revealed significant treatment effects in infarct volume outcomes (one-factor ANOVA; F _(4, 24)_ = 3.16, p = 0.03). Posthoc analyses revealed that the infarct area ratio of stroke rats in 24h group significantly decreased compared to stroke rats in control, 1h, and 6h groups at 7 days after MCAO (control group: 22.1±3.3; 1h group: 20.9±5.1; 6h group: 20.9±3.4; 24h group: 4.6±1.5; 48h group: 9.9±7.0%, at 7 days after MCAO respectively, p’s<0.05, Fig 4C and S2 Data). There was no significant difference of infarct area ratio between rats in 24h and 48h groups (p = 0.41). The infarct area ratio of rats in 48h group showed a trend of reduction in infarct volume compared to control group, 1h group, and 6h group, but there was no statistical differences detected (p = 0.068 vs. control; p = 0.11 vs. 1h; p = 0.099 vs. 6h group; p’s >0.05).

### Correlation between neurological function and infarct volumes

**Fig 5:**
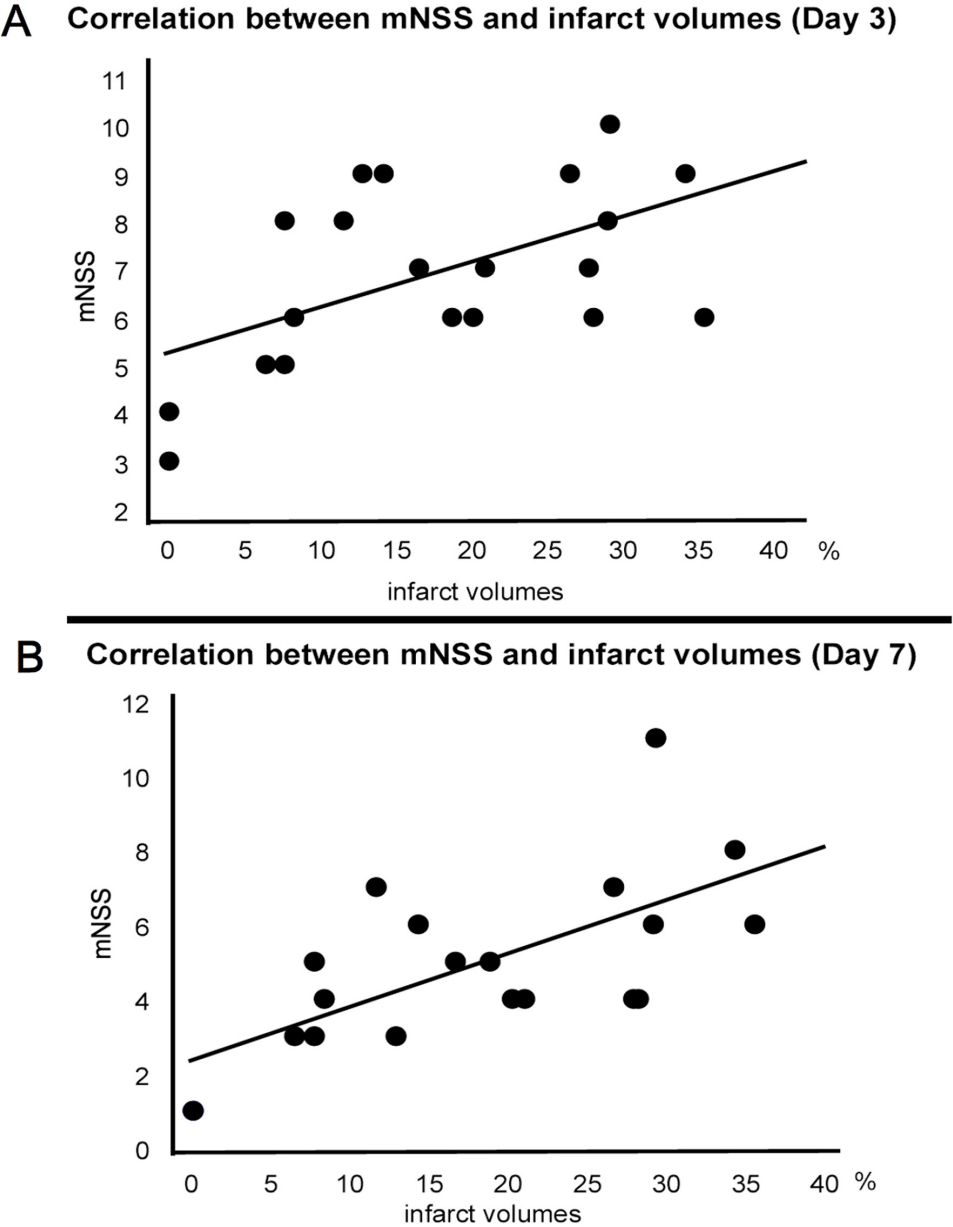
Correlation between mNSS and infarct volumes. The graph demonstrates that improvement in neurological performance as revealed by mNSS at Day 3 (A) and Day 7 (B) positively correlated with reduced infarct volumes.

There was significant correlation between neurological function at 3 and 7 days after MCAO and infarct volumes (3 days after MCAO: Y = 5.283+0.091*X, R^2 = 0.287, correlation efficiency: 0.536, p = 0.0015; 7 days after MCAO: Y = 2.302+0.143*X, R^2 = 0.449, correlation efficiency: 0.670, p = 0.0012, Fig 5).

### Migration of integrated MSCs in vivo

**Fig 6:**
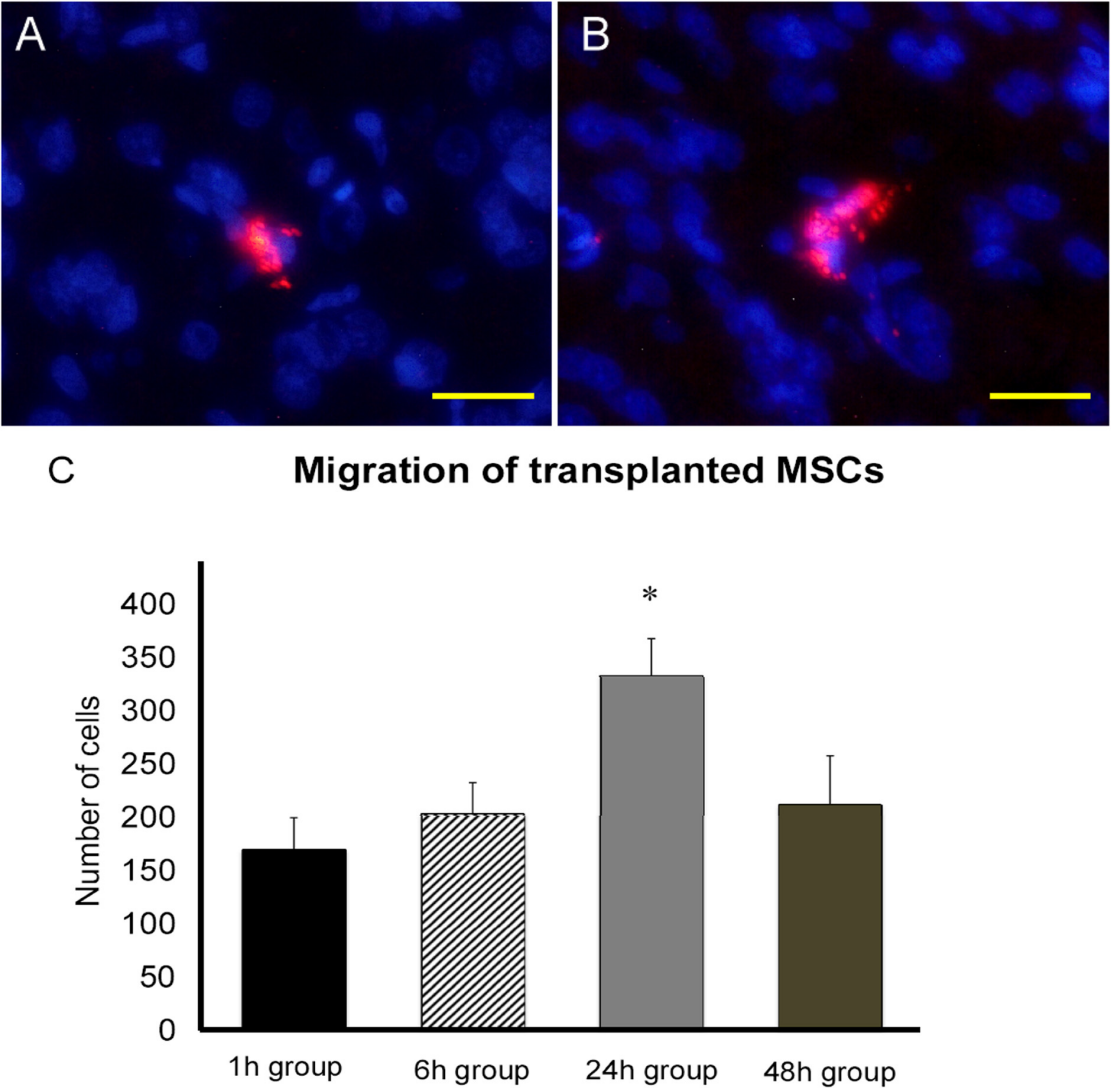
Migration of integrated MSCs in vivo. A, B: Photographs showing integrated MSCs in the brain. Integrated MSCs were mainly detected in the ischemic penumbra at 7 days after MCAO. Scale bar: 20 μm. C: Bar graph showing the number of integrated MSCs in the brain of rats in each group. The number of MSCs was significantly higher in 24h group than other transplantation groups (* p value<0.05, n = 10 in 1h group, 5 in 6h group, 9 in 24h group, 8 in 48h group, respectively).

ANOVA revealed treatment effects on MSC migration (one-factor ANOVA; F _(3, 28)_ = 4,251, p = 0.0135). MSCs were detected in the brain of all transplanted groups at 7 days after MCAO (Fig 6A and 6B). MSCs mainly migrated into the ischemic penumbra. Posthoc analyses revealed that the number of integrated MSCs was significantly higher in stroke rats of 24h group than that of all other groups. (1h group: 170.3±29.7; 6h group: 203.0±29.8; 24h group: 332.9±34.9; 48h group: 212.5±45.5 cells/slice, at 7 days after MCAO respectively, p’s<0.05, Fig 6C and S3 Data).

### Protein assay for neurotrophic factor and chemotactic cytokine

**Fig 7:**
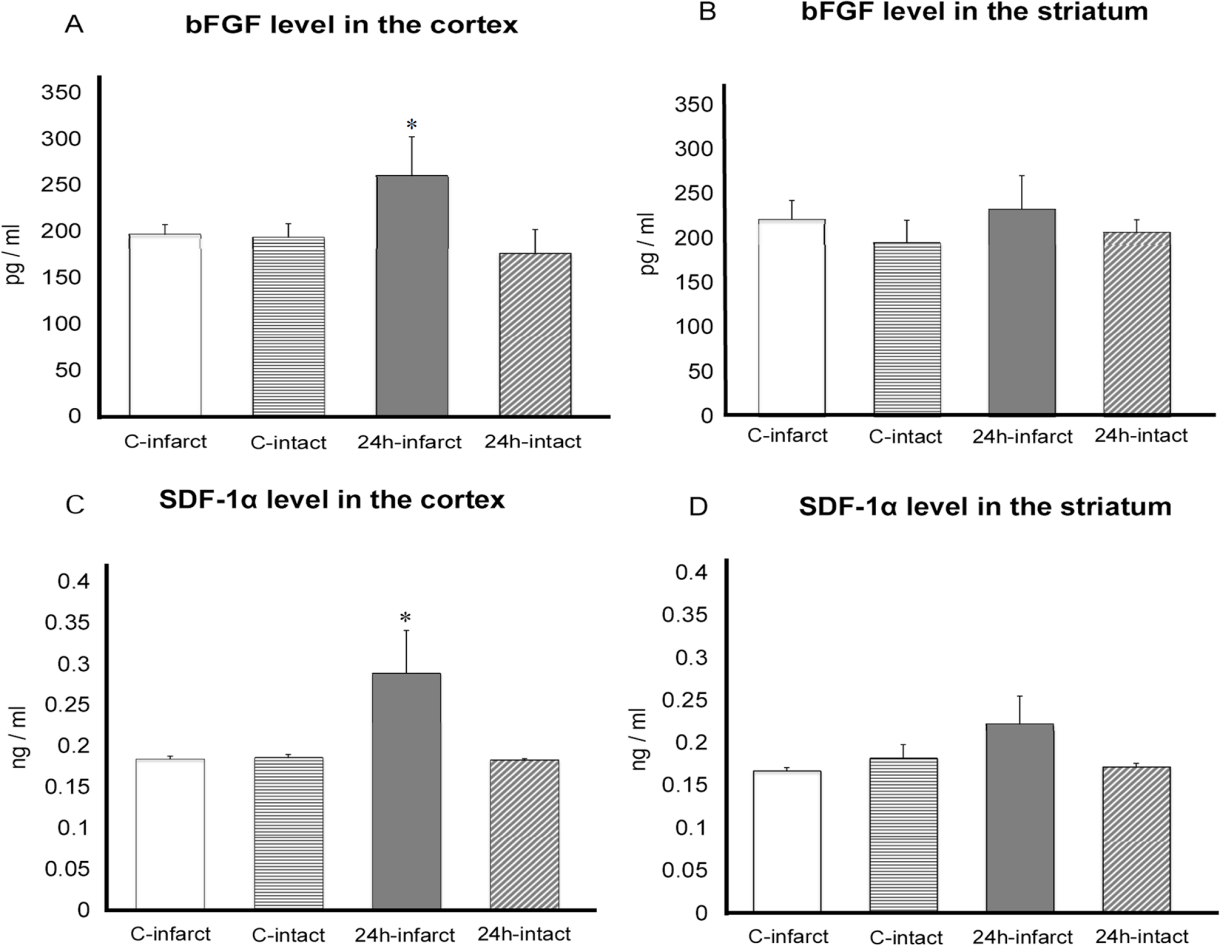
Results of ELISA analyses for bFGF and SDF-1α. A: The bFGF level increased in the infarcted cortex of rats in 24h group. There was significant difference of the bFGF level between the infarcted cortex and the intact cortex of rats in 24h group and the infarcted and intact cortex of rats in control group. B: In the infarcted striatum, bFGF level did not increase by the transplantation (C-infarct: the infarct side of rats in control group; C-intact: the intact side of rats in control group; 24h-infarct: the infarct side of rats in 24h group; 24h-intact: the intact side of rats in 24h group, *p value<0.05, n = 8, respectively). C: SDF-1α level significantly increased in the infarcted cortex of rats in 24h group, compared to all other regions. D: In the infarcted striatum, SDF-1α level did not increase by the transplantation (* p value<0.05, n = 8, respectively).

ANOVA revealed significant treatment effects on bFGF cortical expression (one-factor ANOVA; F _(3, 32)_ = 2.628, p = 0.0361). Posthoc analyses revealed that the bFGF level of the infarcted cortex of stroke rats in 24h group significantly increased compared to the infarcted cortex of rats in control group and the contralateral intact cortex of stroke rats in 24h group and control group at 7 days after MCAO (24h group: infarcted cortex 262.2±37.3, intact cortex 176.3±25.6; control group: infarcted cortex 190.3±9.6, intact cortex 193.3±15.0 pg/ml, respectively, p’s<0.05, Fig 7A and S4 Data). In contrast, ANOVA revealed no significant treatment effects on bFGF striatal expression (one-factor ANOVA; F _(3, 24)_ = 0.395, p = 0.7575) indicating that the bFGF level of the infarcted striatum of stroke rats in 24h group did not increase compared to that of control group and the intact striatum of rats in both groups (Fig 7B and S4 Data).

ANOVA revealed significant treatment effects on SDF-1α cortical expression (one-factor ANOVA; F _(3, 26)_ = 3.658, p = 0.0253). Posthoc analyses revealed that the SDF-1α level of the infarcted cortex of stroke rats in 24h group significantly increased compared to the infarcted cortex of rats in control group and the contralateral intact cortex of stroke rats in 24h group and control group at 7 days after MCAO (24h group: infarcted cortex 0.29±0.052, intact cortex 0.18±0.002; control group: infarcted cortex 0.18±0.004, intact cortex 0.19±0.004 ng/ml, respectively, p value<0.05, Fig 7C and S5 Data). On the other hand, ANOVA revealed no significant treatment effects on SDF-1α striatal expression (one-factor ANOVA; F _(3, 22)_ = 1.609, p = 0.216), indicating that the SDF-1α level of the infarcted striatum did not increase compared to that of control group (Fig 7D and S5 Data).

## Discussion

The present study demonstrated that intra-arterial allogeneic MSC transplantation in ischemic stroke model of rats induced functional recovery with transplantation at 24 hours after MCAO being most effective. The robust behavioral recovery exhibited by stroke rats in the 24h transplanted group was paralleled by reduced infarct volumes and a high number of integrated MSCs successfully migrating towards the ischemic cortex and coinciding with elevated levels of bFGF and SDF-1α in the infarcted cortex.

### Functional effects of intra-arterial MSC transplantation

To date, the efficacy of intravenous MSC transplantation has been well-documented in animal models of ischemic stroke [9, 12, 14, 15, 23, 27, 28]. The neuroprotective effects of intravenous transplantation have been mainly ascribed to secretion of neurotrophic factors but not migration of cells [3, 7, 32]. Recently, intra-arterial MSC transplantation in animal models of ischemic stroke has been similarly shown to induce functional recovery [3, 8, 9, 12, 13, 16, 19, 20, 21]. Interestingly, the migration of intra-arterially delivered cells to the stroke brain has been implicated as a pivotal factor in the observed therapeutic effects. Comparing intra-arterial and intravenous bone marrow mononuclear cells (BMMCs) transplantation following ischemic stroke in rats, results revealed that intra-arterial transplantation resulted in less infarct volumes, better functional recovery, and higher number of BMMCs surviving in the stroke brain than intravenous transplantation [12]. Similarly, a much higher number of human MSCs migrated into the infarct lesion with intra-arterial transplantation than that with intravenous transplantation, further lending support to the notion that the migration of cells to the injured brain contributes to the functional recovery [3]. In the present study, we showed that many integrated MSCs migrated to the infarct lesion, especially in the 24h transplanted group that coincided with functional recovery in stroke rats. Along this line of investigations, intracerebral transplantation could result in higher number of cells deposited into the ischemic brain than intravenous and intra-arterial transplantation [2, 9]. However, intracerebral transplantation is most invasive transplantation method, and there are some risks of hemorrhage or injury of tissue due to transplantation. On the other hand, intravascular transplantation offers a minimally invasive transplantation method. Unfortunately, with intravenous transplantation, many peripherally transplanted cells get trapped in peripheral organs, including the liver, spleen, and lungs [1, 2, 9, 33], indicating that intravenous transplantation does not ensure satisfactory migration of cells into the stroke brain. In this regard, intra-arterial transplantation while also a relatively less invasive intravascular transplantation method, can promote the deposition of a large number of peripherally delivered cells directly into the lesion site. Nevertheless, intra-arterial transplantation carries the risks of cerebral embolism and reduction of cerebral blood flow associated with microstrokes [33]. Similar to intracerebral stereotaxic guided surgical procedure, intra-arterial transplantation may require a certain level of skill to safely and effectively perform the procedure. Despite these limitations, additional studies are warranted to optimize intra-arterial transplantation as a promising transplantation method from the viewpoint of aiding in the successful migration of peripherally integrated cells into the stroke brain.

### Therapeutic window of intra-arterial MSC transplantation

Because the primary cerebral infarct triggers multiple secondary cell death processes in the stroke brain over time, including inflammation, oxidative stress, and endothelial dysfunction, the timing of cell transplantation should be carefully considered [3, 9]. Intra-arterial MSC transplantation at 1 and 4 days after MCAO in rats at led to neuroprotective effects, but transplantation at 7 days after MCAO was less effective [3]. The efficacy of this early transplantation was thought to involve cell migration, reactive astrocyte enhancement, angiogenesis, and reduced inflammatory response. In our study, we further characterized the acute therapeutic time window of intra-arterial transplantation in stroke, and showed that transplantation at 24 hours after MCAO significantly improved neurological function and reduced infarct volume. The highest number of MSCs that migrated to the ischemic brain was also detected in the brain of stroke rats in 24h transplanted group. On the other hand, the number of integrated MSCs was relatively low in 1h and 6h groups, resulting in observable no reduction of infarct volumes. That the supra-acute timing post-stroke was not amenable to MSC migration into the stroke brain suggests that blood-brain-barrier (BBB) may still be intact immediately after cerebral infarct and may be prohibitive to the entry of peripherally delivered MSCs. Indeed, the BBB has been implicated as one of the major limiting factors obstructing MSCs from reaching injured regions of the brain [18, 34]. MSCs delivered by intra-arterial injection need to pass through the BBB before they can successfully migrate and survive into an injured site [18, 35]. Previous studies reported that the approximate time estimate of BBB disruption from onset of ischemia was around 3.8 hours [36, 37]. In view of this observation, the initiation of intra-arterial MSC transplantation after BBB disruption has progressed may be advantageous if the desired goal is to facilitate the engraftment of MSCs towards functional recovery in stroke.

### Neurotrophic factors secreted by transplanted MSCs and their neuroprotective effects

Several neurotrophic factors have been demonstrated to contribute to the therapeutic outcomes of stem cell therapy in experimental stroke. bFGF is one of the most potent neurotrophic factors for the protection of neurons [1, 2, 27, 29, 38]. We previously demonstrated that grafting of encapsulated bFGF-secreting cells protected against ischemic injury after MCAO in rats by directly exerting cytoprotection through suppression of apoptosis in tandem with enhanced angiogenesis at the border of ischemic infarct [38]. Functional recovery after intravenous MSC transplantation in ischemic stroke rats also details the secretion of bFGF from MSCs, which reduces apoptosis and promotes proliferation of endogenous stem and progenitor cells within the peri-infarcted tissue [27, 29]. Intravenous MSC transplantation was reported to promote the production of endogenous bFGF in ischemic stroke model in rats [29]. On the other hand, intravenously transplanted MSCs might be able to secret bFGF in the brain and provide functional recovery [27, 29]. In the present study, bFGF increased in the infarcted cortex of rats in 24h transplanted group, which showed the most robust migration of cells, suggesting that MSCs that migrated to the injured brain secreted bFGF, and in turn reduced lesion volume and contributed to functional recovery. Additionally, SDF-1α also significantly increased in the infarcted cortex of rats in 24h transplanted group. SDF-1α is one of the chemotactic cytokines and the unique ligand for a CXC chemokine receptor (CXCR4) [22, 39]. In addition, SDF-1α may exert neuroprotective effects, in that rats that received intracerebral administration of SDF-1α displayed functional recovery coupled with reduced infarct volumes through upregulation of anti-apoptotic proteins [40]. The expression of SDF-1α increase in the ischemic penumbra due to stroke-induced activation of astrocytes and locally produced SDF-1α induced the migration of intravenously transplanted MSCs into the ischemic hemisphere in ischemic stroke model in rats [40, 41]. On the other hand, several reports suggested that bone marrow stromal cells might secrete SDF-1α [22, 39, 40]. We previously reported that MSCs secreted SDF-1α in vitro and intravenous transplantation of MSCs engineered to secrete SDF-1α led to neuroprotective effects through anti-apoptotic mechanisms in a Parkinson’s disease model of rats [22]. For these reasons, we speculate that the combination of bFGF and SDF-1α likely secreted by the intra-arterially transplanted MSCs, which successfully honed in the ischemic brain, might have contributed to the observed therapeutic outcomes. However, there is a possibility that intra-arterially transplanted MSCs promoted the production of endogenous bFGF and SDF-1α.

### Clinical application of intra-arterial MSC transplantation for ischemic stroke

Clinical studies have been initiated to evaluate stem cell therapy in patients with ischemic stroke. Transplantation of bone marrow mononuclear cells (BMMCs) for ischemic stroke has been initiated [4, 42–44]. Preliminary results [4, 42, 43] have shown that intra-arterial transplantation of autologous BMMCs in ischemic stroke patients at subacute phase was safe and feasible without adverse events, death, and stroke due to procedure in follow up time. In one study [43], patients with moderate to severe ischemic stroke were treated within 3 to 7 days, with no observable complications, and 40% of patients showed good clinical outcome at chronic phase. However, the optimal time window is unclear. Because the condition of patients with ischemic stroke remarkably changes over time, determining the therapeutic window is important for improving stroke treatments. Autologous transplantation in acute phase may be indicated, but in clinical settings the time-consuming expansion of autologous MSCs presents as a challenge, although the safety of intra-arterial cell transplantation has been reported [4, 42, 43]. Alternatively, autologous MSC stem cell banking (e.g., from umbilical cord or placenta) and allogeneic cell transplantation may be options for intra-arterial transplantation in patients with ischemic stroke in acute phase. Clearly, more basic and clinical studies are needed to confirm the safety and efficacy of intra-arterial transplantation of MSCs within the acute time window of stroke.

## Conclusions

This study demonstrates that intra-arterial allogeneic MSC transplantation in rats exposed to transient ischemic stroke model improves neurological function and reduces infarct volumes, likely due to robust migration of peripherally delivered cells into the ischemic brain resulting in upregulated CNS levels of bFGF and SDF-1α. Intra-arterial transplantation at 24 hours after MCAO may be optimal timing for stroke.

## Supporting Information

S1 Data Modified Neurological Severity Score (mNSS) (score). (DOCX)

S2 Data Infarct volumes (%). (DOCX)

S3 Data Migration of integrated MSCs in vivo (number of cells). (DOCX)

S4 Data ELISA analyses bFGF level (pg/ml). (DOCX)

S5 Data ELISA analyses SDF-1α level (ng/ml). (DOCX)
