# Supplementary material for: Intra-Arterial Transplantation of Allogeneic Mesenchymal Stem Cells Mounts Neuroprotective Effects in a Transient Ischemic Stroke Model in Rats: Analyses of Therapeutic Time Window and Its Mechanisms
Source: PLoS One. 2015 Jun 15;10(6):e0127302. doi: 10.1371/journal.pone.0127302 (PMC4468176; doi:10.1371/journal.pone.0127302)
Supplement: S1 Data — (DOCX) [file pone.0127302.s001.docx]

**S1 Data. Modified Neurological Severity Score (mNSS) (Score).**

| Control group |  |  |  |
| --- | --- | --- | --- |
| Number | Score immediately after reperfusion | Score at day3 | Score at day7 |
|  |  |  |  |
| 1 | 8 | 4 | 3 |
| 2 | 11 | 8 | 5 |
| 3 | 11 | 7 | 5 |
| 4 | 10 | 10 | 9 |
| 5 | 10 | 9 | 8 |
| 6 | 9 | 6 | 4 |
| 7 | 11 | 10 | 11 |
| 8 | 11 | 6 | 4 |
| 9 | 10 | 7 | 4 |
| 10 | 11 | 9 | 9 |
| 11 | 11 | 8 | 5 |
| 12 | 11 | 9 | 7 |
| 13 | 10 | 9 | 8 |
| 14 | 9 | 7 | 5 |
| 15 | 10 | 7 | 4 |
| 16 | 11 | 10 | 9 |
| 17 | 10 | 7 | 5 |
|  |  |  |  |
| 1h group |  |  |  |
| Number | Score immediately after reperfusion | Score at day3 | Score at day7 |
| 1 | 11 | 8 | 2 |
| 2 | 10 | 1 | 3 |
| 3 | 10 | 10 | 8 |
| 4 | 10 | 7 | 4 |
| 5 | 10 | 6 | 5 |
| 6 | 10 | 7 | 6 |
| 7 | 10 | 6 | 6 |
| 8 | 9 | 9 | 3 |
| 9 | 10 | 8 | 6 |
| 10 | 10 | 5 | 5 |
|  |  |  |  |
| 6h group |  |  |  |
| Number | Score immediately after reperfusion | Score at day3 | Score at day7 |
| 1 | 9 | 9 | 8 |
| 2 | 10 | 3 | 1 |
| 3 | 11 | 9 | 6 |
| 4 | 10 | 8 | 7 |
| 5 | 10 | 9 | 8 |
| 6 | 10 | 9 | 7 |
| 7 | 11 | 8 | 5 |
| 8 | 9 | 7 | 5 |
| 9 | 8 | 7 | 6 |
| 10 | 10 | 7 | 4 |
| 11 | 10 | 7 | 5 |
|  |  |  |  |
| 24h group |  |  |  |
| Number | Score immediately after reperfusion | Score at day3 | Score at day7 |
| 1 | 10 | 10 | 6 |
| 2 | 11 | 4 | 2 |
| 3 | 11 | 5 | 2 |
| 4 | 11 | 5 | 1 |
| 5 | 10 | 4 | 2 |
| 6 | 11 | 5 | 3 |
| 7 | 10 | 4 | 1 |
| 8 | 11 | 5 | 3 |
| 9 | 10 | 6 | 5 |
| 10 | 10 | 4 | 2 |
| 11 | 10 | 3 | 1 |
| 12 | 10 | 4 | 0 |
|  |  |  |  |
| 48h group |  |  |  |
| Number | Score immediately after reperfusion | Score at day3 | Score at day7 |
| 1 | 10 | 6 | 7 |
| 2 | 10 | 5 | 4 |
| 3 | 8 | 7 | 5 |
| 4 | 10 | 6 | 2 |
| 5 | 11 | 5 | 3 |
| 6 | 11 | 9 | 9 |
| 7 | 9 | 6 | 2 |
| 8 | 10 | 8 | 7 |
| 9 | 10 | 6 | 3 |
| 10 | 9 | 5 | 5 |
| 11 | 9 | 4 | 5 |
| 12 | 9 | 8 | 8 |
