# Supplementary material for: Intra-Arterial Transplantation of Allogeneic Mesenchymal Stem Cells Mounts Neuroprotective Effects in a Transient Ischemic Stroke Model in Rats: Analyses of Therapeutic Time Window and Its Mechanisms
Source: PLoS One. 2015 Jun 15;10(6):e0127302. doi: 10.1371/journal.pone.0127302 (PMC4468176; doi:10.1371/journal.pone.0127302)
Supplement: S2 Data — (DOCX) [file pone.0127302.s002.docx]

**S2 Data. Infarct volumes (%).**

| Number | Control group | 1h group | 6h group | 24h group | 48h group |
| --- | --- | --- | --- | --- | --- |
| 1 | 28.2 | 8.3 | 22.6 | 7.6 | 0 |
| 2 | 29.3 | 18.8 | 14.2 | 0 | 41.9 |
| 3 | 20.2 | 35.5 | 11.5 | 6.5 | 17.4 |
| 4 | 20.9 | 12.7 | 32.7 | 0 | 0 |
| 5 | 26.5 | 29.1 | 16.5 | 5.5 | 0 |
| 6 | 7.7 |  | 27.8 | 8.3 | 0 |
