# Supplementary material for: Intra-Arterial Transplantation of Allogeneic Mesenchymal Stem Cells Mounts Neuroprotective Effects in a Transient Ischemic Stroke Model in Rats: Analyses of Therapeutic Time Window and Its Mechanisms
Source: PLoS One. 2015 Jun 15;10(6):e0127302. doi: 10.1371/journal.pone.0127302 (PMC4468176; doi:10.1371/journal.pone.0127302)
Supplement: S3 Data — (DOCX) [file pone.0127302.s003.docx]

**S3 Data. Migration of integrated MSCs in vivo (number of cells).**

| Number | 1h group | 6h group | 24h group | 48h group |
| --- | --- | --- | --- | --- |
| 1 | 113.3 | 252.4 | 245.2 | 180 |
| 2 | 114.8 | 231.4 | 392.4 | 400.5 |
| 3 | 341.9 | 195.2 | 392.4 | 299.4 |
| 4 | 120.1 | 90.5 | 287.1 | 335.7 |
| 5 | 110 | 245.7 | 492.5 | 253.8 |
| 6 | 120 |  | 280.5 | 83.3 |
| 7 | 247.1 |  | 331 | 71.9 |
| 8 | 301 |  | 426.2 | 75.7 |
| 9 | 173.3 |  | 149.1 |  |
| 10 | 61.9 |  |  |  |
