# Supplementary material for: Intra-Arterial Transplantation of Allogeneic Mesenchymal Stem Cells Mounts Neuroprotective Effects in a Transient Ischemic Stroke Model in Rats: Analyses of Therapeutic Time Window and Its Mechanisms
Source: PLoS One. 2015 Jun 15;10(6):e0127302. doi: 10.1371/journal.pone.0127302 (PMC4468176; doi:10.1371/journal.pone.0127302)
Supplement: S4 Data — (DOCX) [file pone.0127302.s004.docx]

**S4 Data. ELISA analyses bFGF level (pg/ml).**

| Control group | Number | Infarcted cortex | Intact cortex | Infarcted striatum | Intact striatum |
| --- | --- | --- | --- | --- | --- |
|  | 1 | 215.1164 | 194.6393 | 309.3893 | 228.6434 |
|  | 2 | 190.1323 | 180.9558 | 216.6081 | 205.8311 |
|  | 3 | 205.7552 | 192.6852 | 234.3328 | 221.5317 |
|  | 4 | 172.1329 | 204.2870 | 114.0347 | 155.0094 |
|  | 5 | 131.4264 | 248.3017 | 234.1140 | 301.9493 |
|  | 6 | 197.2932 | 210.5624 | 208.2932 | 162.4117 |
|  | 7 | 210.4322 | 163.5388 | 228.1274 | 84.2698 |
|  | 8 | 198.8617 | 149.2897 | 244.0206 | 181.8806 |
| 24h group | Number | Infarcted cortex | Intact cortex | Infarcted striatum | Intact striatum |
|  | 1 | 281.6682 | 210.5041 | 82.9070 | 179.7364 |
|  | 2 | 408.3992 | 156.1582 | 273.5022 | 155.4470 |
|  | 3 | 231.2234 | 119.6993 | 309.1157 | 235.0440 |
|  | 4 | 331.1622 | 112.1893 | 168.6858 | 236.4117 |
|  | 5 | 248.0375 | 242.7394 | 344.5651 | 164.4735 |
|  | 6 | 265.5839 | 202.5505 | 140.6658 | 241.3900 |
|  | 7 | 195.9726 | 185.0908 | 306.7472 | 231.8313 |
|  | 8 | 135.4713 | 180.9288 | 270.1099 | 212.6318 |
