# Supplementary material for: Intra-Arterial Transplantation of Allogeneic Mesenchymal Stem Cells Mounts Neuroprotective Effects in a Transient Ischemic Stroke Model in Rats: Analyses of Therapeutic Time Window and Its Mechanisms
Source: PLoS One. 2015 Jun 15;10(6):e0127302. doi: 10.1371/journal.pone.0127302 (PMC4468176; doi:10.1371/journal.pone.0127302)
Supplement: S5 Data — (DOCX) [file pone.0127302.s005.docx]

**S5 Data. ELISA analyses SDF-1α level (ng/ml).**

| Control group | Number | Infarcted cortex | Intact cortex | Infarcted striatum | Intact striatum |
| --- | --- | --- | --- | --- | --- |
|  | 1 | 0.1812 | 0.1847 | 0.1612 | 0.1446 |
|  | 2 | 0.1843 | 0.1973 | 0.1656 | 0.1346 |
|  | 3 | 0.1747 | 0.1843 | 0.1569 | 0.1630 |
|  | 4 | 0.1821 | 0.1956 | 0.1791 | 0.2023 |
|  | 5 | 0.1725 | 0.1783 | 0.1577 | 0.2291 |
|  | 6 | 0.1882 | 0.1847 | 0.2500 | 0.1221 |
|  | 7 | 0.1804 | 0.1708 | 0.1791 | 0.1782 |
|  | 8 | 0.2095 | 0.1837 | 0.1813 | 0.1590 |
| 24h group | Number | Infarcted cortex | Intact cortex | Infarcted striatum | Intact striatum |
|  | 1 | 0.1838 | 0.1878 | 0.1604 | 0.1604 |
|  | 2 | 0.5509 | 0.1812 | 0.3683 | 0.1712 |
|  | 3 | 0.1834 | 0.1695 | 0.1643 | 0.1769 |
|  | 4 | 0.4570 | 0.1860 | 0.1743 | 0.1643 |
|  | 5 | 0.1704 | 0.1799 | 0.1586 | 0.1569 |
|  | 6 | 0.1869 | 0.1869 | 0.1899 | 0.1730 |
|  | 7 | 0.3535 | 0.1847 | 0.3722 | 0.1812 |
|  | 8 | 0.2278 | 0.1882 | 0.1860 | 0.1904 |
